# A new microbial gluten-degrading prolyl endopeptidase: Potential application in celiac disease to reduce gluten immunogenic peptides

**DOI:** 10.1371/journal.pone.0218346

**Published:** 2019-06-27

**Authors:** María de Lourdes Moreno Amador, Miguel Arévalo-Rodríguez, Encarnación Mellado Durán, Juan Carlos Martínez Reyes, Carolina Sousa Martín

**Affiliations:** 1 Departamento de Microbiología y Parasitología, Facultad de Farmacia, Universidad de Sevilla, Sevilla, Spain; 2 Biomedal S.L., Sevilla, Spain; Tulane University, UNITED STATES

## Abstract

Gluten is a complex of proteins present in barley, wheat, rye and several varieties of oats that triggers celiac disease in genetically predisposed subjects. Gluten is notoriously difficult to digest by mammalian proteolytic enzymes and therefore, proline-rich digestion-resistant peptides contain multiple immunogenic epitopes. Prolyl endopeptidases (PEP) hydrolyse internal proline residues on the carboxyl side of peptides and have been proposed for food gluten detoxification and as oral enzyme supplementation for celiacs. The aim of this study was to identify new gluten-degrading microbial enzymes with the potential to reduce gluten immunogenicity by neutralizing its antigenic epitopes. Using a gluten-degrading colony screening approach, a bacterial isolate (2RA3) displaying the highest glutenase activity was selected, characterized and its genome completely sequenced. The identification through 16S rDNA gene sequencing showed a 99,1% similarity to *Chryseobacterium taeanense*. Hydrolysis of gluten immunogenic peptides (GIP) was further monitored, over a 48-hour period, by colony encapsulation in gliadin-containing microspheres, followed by detection with the G12 anti-GIP monoclonal antibody. Glutenase activity was detected in the extracellular medium of 2RA3 cultures, where gel electrophoresis and gliadin zymography revealed the presence of a ~50 kDa gluten-degrading enzyme. Nano-ESI-Q-TOF of the excised active band identified 7 peptides contained in the protein product predicted for an open reading frame (ORF) in the 2RA3 genome. Based on sequence similarity to the PEP family, the new enzyme was named PEP 2RA3. The PEP 2RA3 coding sequence was PCR-amplified from *C*. *taeanense* 2RA3, cloned and expressed in *Escherichia coli* as a C-terminally His-tagged recombinant protein and purified by Ni-NTA affinity chromatography. The recombinant protein, with predicted molecular mass and isoelectric point of 78.95 kDa and 6.8, respectively, shows PEP activity with standard chromogenic substrates, works optimally at pH 8.0 and 30°C and remains stable at pH 6.0 and 50°C, indicating a potential use in gluten-containing food process applications. The ability of the recombinant enzyme to degrade GIP in beer into smaller peptides was confirmed.

## Introduction

Gluten is the common name for proteins present in all cereals of the *Triticeae* grass tribe in which the major industrially relevant crops are barley, wheat and rye [1]. A high content of proline and glutamine residues is found in gluten proteins, which makes them resistant to gastrointestinal track and potentiates their deamidation by tissue transglutaminase [2–3].

Gluten-related disorders including celiac disease (CD), wheat allergy, baker’s asthma and non-celiac gluten sensitivity, which have gradually emerged as an epidemiologically significant phenomenon with a relatively high global prevalence (5–10% of the population) [4–5]. Celiac disease develops in genetically susceptible individuals and is triggered by the exposure to partially digested gluten proteins [6]. To date, the only available therapy for CD is the complete avoidance of dietary gluten. However, sustaining a strictly gluten-free diet (GFD) is very challenging. The rates of adherence to GFD described in the literature vary considerably (17% to 80%) depending on factors such as the patient’s age, the age at the time of CD diagnosis, self-reporting, and laboratory testing [7–9]. Although gluten levels in individual products have been determined, the amount of gluten in a “gluten-free” diet as consumed by people with CD remains unknown. Therefore, there is a need for the use of enzymes as additives or as processing aids in the food biotechnology industry, either to detoxify gluten or as non-dietary oral therapies for celiac patients [10].

Proteolysis-resistant gluten peptides account for most of the immunotoxic reactions in T cells of CD patients. Furthermore, mammalian digestive enzymes are not easily available for the proteolytic degradation of protease-resistant domains in gluten due to appear to require enzymatic cleavage specificities [8, 11]. Therefore, microbial prolyl endopeptidases (PEPs, also known as prolyl oligopeptidases) may constitute the therapeutic keys for the treatment of CD since they are especially effective in the hydrolysis of peptide bonds on the carboxyl side of internal proline residues in gluten-derived oligopeptides [12]. PEPs belong to serine protease family (clan SC, family S9), which is a group of peptidases can hydrolyze peptides smaller than 30 residues. Although membrane bound form of PEP enzyme has been characterized, it is generally reported to be cytosolic [13]. PEPs are structurally and functionally closely related to that of the dipeptidyl peptidase IV (DPP-IV), oligo peptidase B and acyl-aminoacyl peptidase sub-families [14].

Prolyl endopeptidases from *Flavobacterium meningosepticum*, *Sphingomonas capsulate* and *Myxococcus xanthus* have shown their potential in pharmaceutical use, since they are able to successfully degrade immunogenic gluten amino acid sequences [12]. A combination of aspergillopepsin from *Aspergillus niger* and DPP-IV from *Aspergillus oryzae*, was found to successfully degrade small amounts of gluten *in vitro* [15]. A mixture of barley cysteine endoprotease EP-B2 and the PEP from *Sphingomonas capsulata*, designated in clinical trials as ALV003, has been shown to successfully degrade immunogenic gluten fragments in the stomach [16–17] and a synthetic enzyme called KumaMax has produced similar *in vitro* results to those of ALV003 [18], but remains under development. Further to these enzymes, other dietary supplements rely mainly on DPP-IV are available on the market to help in removing the gluten toxicity despite their limited activity [19].

Here we report on the isolation and identification of the significantly highest gluten-degrading species with respect to those of reference in the literature, with the potential to neutralize the gluten immunogenic peptides (GIP) by disrupting their antigenic epitopes. In this study, recombinant expression of the *Chryseobacterium taeanense* 2RA3 PEP enzyme in *E*. *coli* and purification with nickel-chelate affinity chromatography was carried out. Complete characterization of the purified enzyme comprised determinations of molecular weight, isoelectric point, optimal temperature and pH values, substrate specificity and the ability to degrade standard chromogenic substrates and beer gluten.

## Materials and methods

### Sample collection and site description

Samples were collected from the rhizosphere associated with cereal crops in Isla Mayor, Seville (Spain). The Spanish Rice Federation issued the permission for the study in Hato Ratón zone. The physico-chemical characteristics of the field soil were as follows: salinity 60 mM, pH 8.26, 58.5% clay and 1.96% organic matter. The roots were first deposited in clean plastic containers and washed with tap water to remove any attached soil. The roots were then immersed in 70% ethanol for 3 min and washed five times with sterile distilled water before stirring them in a saline solution (0.9% NaCl, w/v) for 1 hour at 28°C. Samples of the solution were then plated on appropriate selection media.

### Bacterial strains and plasmids

Reference strains for PEP activity were purchased from the Spanish Type Culture Collection and included: *F*. *meningosepticum*-CECT 447^T^, *S*. *capsulata*-CECT 4388^T^, *M*. *xanthus*-CECT 422^T^ and *E*. *coli*-CECT 434^T^. Two reference strains from the German Collection of Microorganisms were included for comparison in the classification study: *C*. *taeanense*-DSM 17071^T^ and *Chryseobacterium taichungense* (*C*. *taichungense*-DSM 17453^T^). *E*. *coli* strain REG-811 and the commercial vector pALEX2-HCa were supplied by Biomedal (Seville, Spain).

### Glutenase activity

Samples obtained from roots as described above were examined for the presence of culturable bacteria with glutenase activity. To this end, samples were inoculated onto a Tryptic Soy Agar (TSA) medium with cycloheximide (100 μg/ml) as a fungal growth inhibitor and 0.1% w/v gliadin (standard concentration) as the glutenase substrate. Plates were incubated at 37°C for 72 hours aerobically. Zones of clarification of gliadin around colonies appearing over the next 48 to 72 hours were considered as evidence of glutenase activity. In order to optimize the gluten degradation activity, various concentrations of gliadin (0.04, 0.05, 0.06, 0.07, 0.1, 0.2, 0.3, 0.4 and 0.5%, w/v) were tested in the TSA medium.

### Bacterial identification and culturing

Standard phenotypic tests were performed according the protocols described in reference taxonomy studies [20–22] comprising catalase and oxidase production, Gram reaction, motility, H_2_S production, utilization of carbon sources and hydrolysis of various substrates among others [20–22]. Cell morphology in exponentially growing cultures was carried out by means of light microscopy (model CX 31 2; Olympus). The strain was grown on plates with TSA and incubated at 28°C in an orbital shaker (New Brunswick Scientific Co.) at 200 rpm for maintenance and routine culture.

The 16S rRNA gene was amplified [23] and sequenced using an automated DNA sequencer model 9700 (Applied Biosystems). The total 16S rRNA gene sequence was aligned to the most similar 16S rRNA gene sequences available from the databases in order to construct the phylogenetic trees using the neighbour-joining algorithm with the molecular evolutionary genetics analysis (MEGA) v.4.0 and 1000 replicates for bootstrap analysis [24].

### DNA-DNA hybridization

As described by De Ley [25], DNA-DNA hybridization was carried out under consideration of the modifications described by Huss [26] using a model Cary 100 Bio UV/VIS-spectrophotometer equipped with a Peltier-thermostated 6x6 multicell changer and a temperature controller with an in situ temperature probe (Varian). The G+C content of the genomic DNA was also determined [27].

### Culture conditions of strain 2RA3

In order to determine the salinity growth range of strain 2RA3, medium Tryptone Soya Broth (TSB) was used with increasing levels of NaCl (0, 0.5, 1, 3, 5, 7, 10, 15, 20 and 25% w/v). TSB medium was used to explore the temperature and pH growth range through the increasing temperatures (28, 37, 45 and 55°C) and pH values (4, 5, 6, 7, 8, 9 and 10). Monitoring the growth curves of strain 2RA3 were obtained by using a Perkin-Elmer spectrophotometer at various incubation times at 600 nm. For this purpose, 20 mL tubes containing 5 mL of TSB medium were each inoculated with 100 μL of a stationary-phase culture and incubated at 28°C and 200 rpm. Tubes of samples were taken after 12, 18, 24, 40 and 48 hours in order to monitor the culture absorbance.

### Cell fractionation

Strain 2RA3 cells from 24 h cultures were centrifuged at 10,000 g (Sorvall Evolution RC) for 20 min at 4°C. The culture supernatant was reserved for the determination of the extracellular enzyme activity. The pellet was washed in a 25 mM phosphate buffer (pH 7.0) and cell disruption by ultrasonic treatment (Labsonic, Braun Biotech International) was performed for 4 min (50%) and centrifuged at 10,000 g for 10 min tat 4°C o remove cellular debris. The supernatant was then kept as the intracellular fraction and stored at -20°C prior its use.

### Immunological assays

Cellena Flow Focusing technology was used for the microencapsulation of strain 2RA3 cells and was managed to produce monodisperse alginate gliadin-microparticles containing individual bacteria. Cell proliferation resulting in microcolony formation was monitored in a Complex Object Parametric Analyzer and Sorter (COPAS) SELECT flow cytometer (Union Biometrica). Relative microcolony size was monitored by measuring the time of flight, the optical extinction and fluorescence. The time of flight minimum was fixed at 150, and the extinction signal was 3.1. Sheath fluid pressure was adjusted to 4.40–5.20, whereas sample fluid pressure was set to maintain a frequency of 15–25 events per second. Size and shape monodisperse alginate microcapsules, containing individual cells, were reproducibly obtained ranging from less than 100 μm to over 600 μm.

Hydrolysis of gliadin content was detected by means of combining the particles with the G12 monoclonal antibody (moAb) conjugated to Fluorescein IsoTiocyanate (FITC) and monitored after 96 hours of incubation.

A commercial lateral flow test (LFT) kit based on moAb G12 (GlutenTox, Biomedal, S.L., Spain) was used to analyse the location of glutenase activity. A mixed gliadin stock solution of 2 mg/ml was prepared in 60% ethanol (v/v). An aliquot of the gliadin stock solution was added to the microbial cell fractions to reach a final gliadin concentration of 90 and 60 ppm and the mixture were incubated for 2 hours. Samples were diluted (1:10 to 1:300) in the buffer solution provided by the manufacturer. GlutenTox sticks were dipped into the reaction solution (300 μl) for 10 min before being removed and allowed to air dry.

### Gel electrophoresis

Sodium dodecyl sulphate-polyacrylamide gel electrophoresis (SDS-PAGE) was conducted as described by Laemmli [28]. In the study SDS-PAGE was usually performed with gels of 10% (w/v) of acrylamide according to the manufacturer´s recommendations and low-molecular-weight proteins (Pharmacia Biotech) were used as markers. Native PAGE (4–20% w/v of polyacrylamide) was employed and high-molecular-weight proteins (Pharmacia Biotech) were used as markers. Gels were run at 120 V for 16 h at 4°C. In accordance with native and SDS-PAGE, the proteins were stained with Coomassie blue (0.1%, w/v).

### Gliadin zymography

Zymographic analysis for detection of glutenase activity was performed in SDS-PAGE using gliadin (0.1%, w/v) as a substrate. After protein separation, the SDS was removed from the gel by soaking them for 30 min in Triton X-100 (2.5% w/v) at room temperature. The gel was then incubated in a buffer (50 mM Tris-HCl, pH 7.5, 5 mM CaCl_2_) overnight. The gel was stained in a solution of 0.1% (w/v) Coomassie Blue in acetic acid:methanol:water (10:30:60) for 1 hour and distained in acetic acid:methanol:water (10:30:60).

### Protein identification of the glutenase

Proteins with glutenase activity were identified through the aforementioned gliadin zymography. Briefly, protein spots of interest were manually excised from stained SDS/PAGE gels, placed in an Eppendorf tube, and washed twice with double-distilled water. Analysis was performed in the Proteomics facility of the Proteomics Unit at the Príncipe Felipe Research Centre.

C18 PepMap guard column (300 μm × 5 mm 5 μm, 100 Å, LC Packings, The Netherlands) was used to pre-concentrate the samples in 0.1% formic acid (FA) at 30 μl/min for 3 min. Then, elution in a C18 PepMap (75 μm × 50 cm, 3 μm, 100 Å, LC Packings, The Netherlands) was held using a 90 min linear gradient from 5 to 55% 0.1% FA in 95% acetonitrile (ACN).

The eluent was sprayed into a nano-ESI-Q-TOF mass spectrometer (Qstar XL system, Applied Biosystems, Framingham, MA, USA) with a nanospray source of the mass spectrometer, and information-dependent acquisition analysis was carried out with acquisition cycles in mass spectrometry (MS) and MS/MS mode throughout the whole chromatogram. The proteomics analyser (Applied Biosystems) was employed in 1-kV ion-reflector mode and selected the five most intense precursors in each fraction with Collision-induced dissociation (CID). The MS/MS information was sent to the MASCOT server (http://www.matrixscience.com) using the MASCOT DAEMON software (Matrix Science, London, UK). The proteins with a score higher than the homology or the significance threshold were identified with a confidence ≥ 95%.

### Bioinformatical analysis of prolyl endopeptidase genes

Predicted protease genes to be proline-specific PEP were used as a search term to screen the full-length sequences of the *Chryseobacterium taeanense* 2RA3 genome. Sequences were identified following a tBLASTn [29] search of the National Center for Biotechnology Information (NCBI) (http://www.ncbi.nlm.nih.gov/Blast) GenBank databases [30]. Open reading frames (ORFs) were identified using the program ORF Finder (http://www.ncbi.nlm.nih.gov/gorf/gorf.htlm). Conserved domains and motifs were determined using the Conserved Domain Database (http://www.ncbi.nlm.nih.gov/Structure/) [31]. Protein analyses were performed with various programs from the ExPASy (Expert Protein Analysis System) of the Swiss Institute of Bioinformatics. The physicochemical properties of proteins were explored by ProtParam, including the molecular weight, theoretical isoelectric point (pI), amino acid composition, extinction coefficient, aliphatic index, instability index, grand average of hydropathicity (GRAVY), and total number of positively and negatively charged residues. The alignments were performed using DNASTAR Lasergene Software and the Clustal W program of EBI (European Bioinformatics Institute).

### Cloning and expression of prolyl endopeptidase genes

The PEP gene was amplified from the genomic DNA from *Chryseobacterium taeanense* 2RA3. Oligonucleotides used for PCR amplification included: PEPF (5’-ATGAAATTCAGACCAATATTACTAACC-3’) and PEPR (5’-TTGGTACCTTTAAATTTTTAATCCCCATTTCAA-3’). The gene was cloned inside the expression vector pALEX2-HCa (Biomedal, Spain) where it was flanked by an in-frame N-terminal histidine-tag. The pALEX2-HCa-PEP-2RA3 vector was transformed into *E*. *coli* REG-811 cells (Biomedal, Spain) and selected on LB medium plates containing 100 μg/ml ampicillin/kanamycin. Cultures were grown aerobically at 37°C to an optical density at 600 nm of approximately 0.8–1.0, when the gene expression was induced with 1 mM salicylate and 10 mM methylbenzoate and incubated overnight at 20°C. Cells were harvested by centrifugation at 4°C (5,000 g, 10 min) and suspended in 20 ml of potassium phosphate pH 8.0. Cell suspension was passed through a French press at a pressure of 800 bar and subsequently centrifuged for 15 min at 10,000 g.

### Purification of PEP

The protein-containing fraction was dissolved in a PBS (pH 7.0) buffer and centrifuged at 10,000 g for 15 min at 4°C and applied to a Ni-NTA resin (ABT, Spain) pre-equilibrated according to the manufacturer´s recommendations. The column was washed with 20 mM NaH_2_PO_4_, 400 mM NaCl, 5 mM imidazole, pH 8.0 (soluble protein wash buffer). Bound proteins were subsequently eluted by gradually increasing the imidazole molarity (25, 50, 75, 100, 125, 200, 250, 300 and 500 mM). The elution fractions were analyzed on reducing SDS-PAGE, and fractions with the presumed desired protein were pooled and dialyzed with a 3500 MWCO Slide-A-Lyzer^®^ Dialysis Cassette (Thermo Fisher Scientific, Ireland). Protein concentration was estimated by means of a BCA protein assay kit (Thermo Scientific) in accordance with the manufacturer’s instructions.

### Prolyl endopeptidase activity assays

Prolyl endopeptidase activity was fluorimetrically assayed by monitoring cleavage of synthetic fluorogenic peptides Z-Gly-Pro-pNA and Suc-Ala-Pro-pNA (Z-, benzyloxycarbonyl-; -pNA, -p-nitroanilide; Suc-, succinyl-) (Bachem, Torrance, CA, U.S.A.). Z-Gly-Pro-pNA was dissolved in a PBS/water/dioxane (8:1.2:0.8, v/v) assay mixture [12]. The concentration of Z-Gly-Pro-pNA was varied from 100–600 μM and Suc-Ala-Pro-pNA from 100–3000 μM.

Hydrolysis of Suc-Ala-Pro-pNA was monitored in a reaction mixture (300 μl) consisting of 30 μl of 10×PBS buffer, a final concentration of 0.2 μM enzyme, and Suc-Ala-Pro-pNA (5 mM stock) at final concentrations between 100 μM and 3 mM. The release of the pNa was spectrophotometrically detected at a wavelength of 410 nm.

For measurement of the influence of pH on the enzyme activity, a series of pH buffer solutions were prepared using citric acid/ NaOH (pH 2–6), Tris/HCl (pH 6–8), and glycine/NaOH (pH 8–12). Reaction mixtures (300 μl) consisted of 30 μl of 10×pH buffer, final concentration of 0.02 μM enzyme and, 1 M Suc-Ala-Pro-pNA.

### Western blot analysis

SDS-PAGE was performed under conditions above mentioned. Proteins beer fractions in the gel were stained with silver staining and transferred to a polyvinylidene fluoride (PVDF) membrane. The PVDF membranes were incubated with G12. After washing, anti-mouse immunoglobulin G phosphatase antibody (Sigma) was added.

## Results and discussion

### Screening of gluten-degrading bacteria

**Fig 1:**
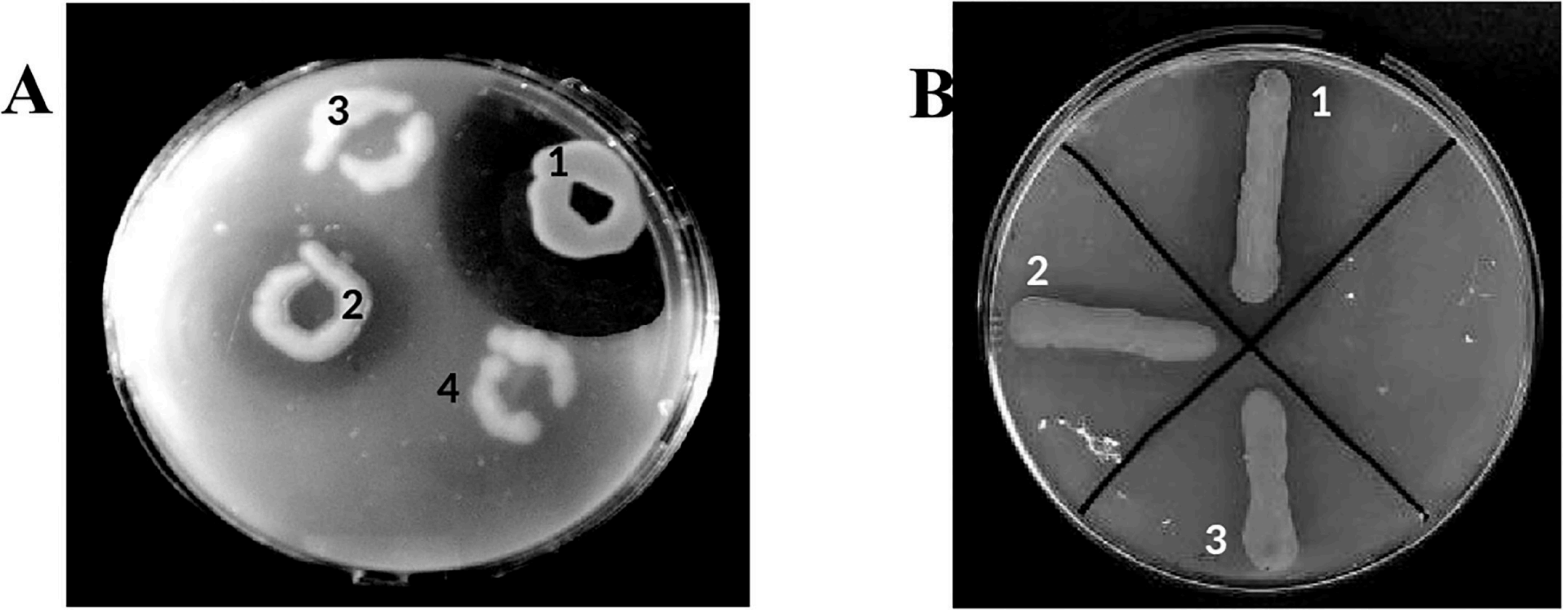
Comparison of glutenase activity of the 2RA3 strain with a reference gluten degrader in TSA medium with gliadin (0.1% w/v). **A**. 1, strain 2RA3; 2, *Flavobacterium meningosepticum*; 3 and 4, *Escherichia coli* (negative control). **B**. 1, strain 2RA3; 2, *Myxococcus xanthus*; 3, *Sphingomonas capsulata*.

Most of the microorganisms in the rhizosphere soil are related to plant species with enzymes that can efficiently hydrolyse and solubilize substrates unavailable to plants and markedly increase their growth [32]. Using the selective plating approach, rhizosphere-soil was screened for gluten-degrading bacteria. We set 0.1% (w/v) gliadin in the target to search for enzymes with improved gluten-detoxification potential. Twenty-three colonies showing glutenase activity were obtained. Gluten-degrading activity was detected by clear zones hydrolysis around the colonies growing on gliadin-containing agar plates. From among the many strains, strain 2RA3 was selected for its ability to produce the highest activity on plates with a larger gliadin-clear zone. Notwithstanding its efficacy, PEPs from various extensively studied reference gluten-degrading bacteria (*Flavobacterium meningosepticum*, *Myxococcus xanthus*, and *Sphingomonas capsulata*) were employed to compare their activities with the selected 2RA3 strain. As indicated in Fig 1A and 1B, PEP from the strain 2RA3 showed a clear hydrolysis zone significantly greater than that produced by the reference strains. In order to test the optimal gliadin concentration for the bacterial activity the selected strain was inoculated onto plates containing increasing concentrations of gliadin ranging from 0.04 to 0.5% (w/v) since it has been reported that the concentration of certain antigenic peptides builds up (possibly in the range of 0.1 mM) in the upper intestinal lumen following ingestion of a typical meal harbouring ∼10 g of gluten [33]. The strain 2RA3 was able to hydrolyse all tested gliadin concentrations, whereby 0.05% gliadin was the optimum concentration without affecting the growth of the bacteria.

### Evaluation of the bacterial reduction of gluten immunogenic peptides

Although growth and gliadin hydrolysis plating approach is an appropriate initial step for the selection of microorganisms capable of utilizing gluten, highly active gluten-degrading enzymes nor specificities of interest targeting immunogenic domains are not necessarily produced and exhibit by the isolated strains [34]. Therefore, further evaluation was required to check the reduction of gluten immunogenic peptides (GIP) by the selected strain since the detection of GIP has been proposed as the analytical standard reference for the determination of the immunopathological risk of gluten exposure in gluten-related diseases [35].

**Fig 2:**
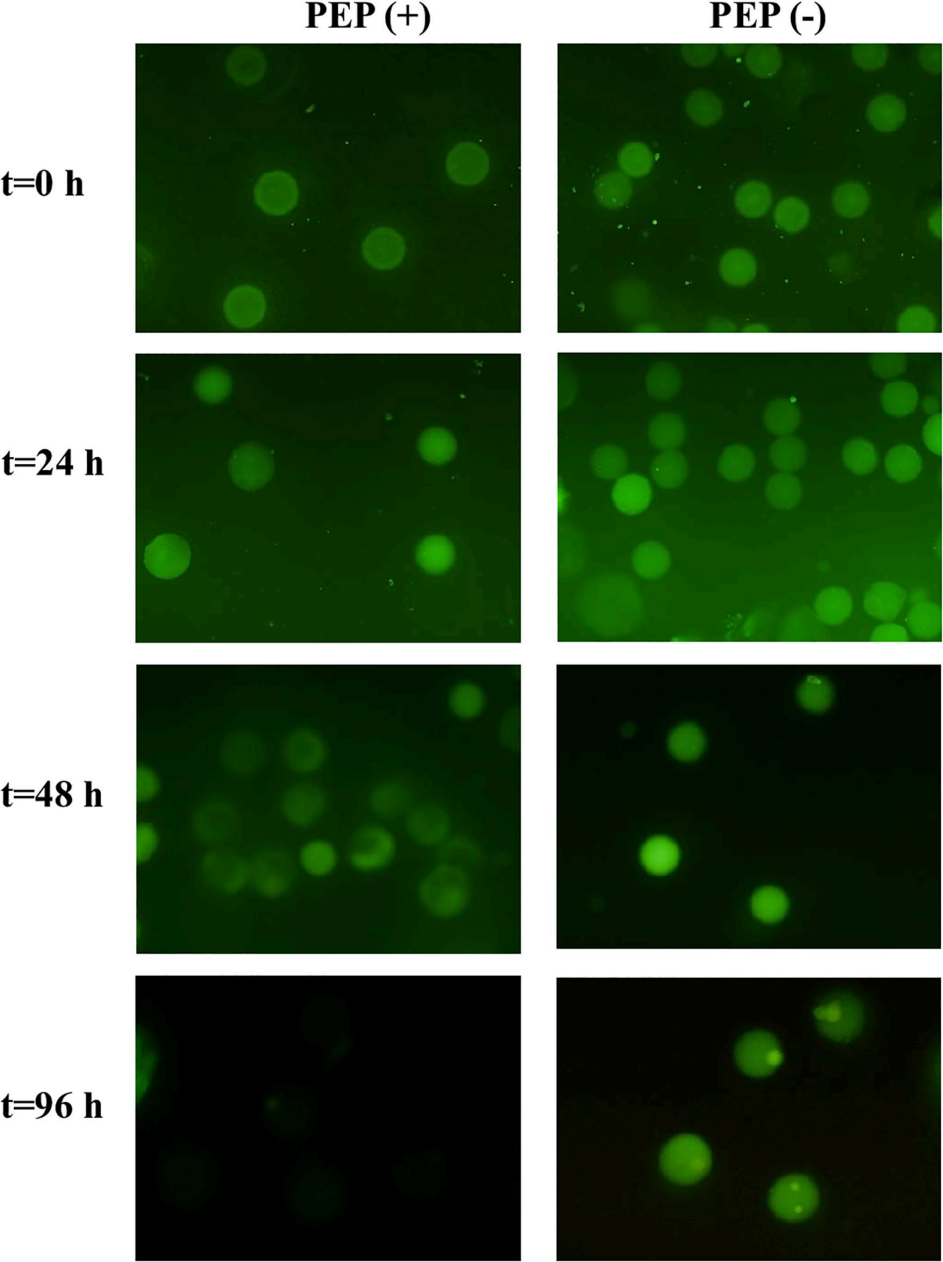
Detection of glutenase activity in microencapsulated bacteria. Fluorescence intensity of each individual alginate particle was detected and quantified with the moAb G12-FITC at different incubation times. Micrographs corresponding to the most representative samples are shown. PEP (+) show the fluorescence of microencapsulated bacteria hydrolyzing the gliadin, and PEP (-), containing microparticles without bacteria.

Previous reports have demonstrated that the G12 moAb specifically recognizes the immunoreactive peptides in those gluten proteins that have a reported characterization of the consistency with *ex-vivo*-quantified immunogenicity with T-cells from celiac individuals [35–39]. To monitor GIP hydrolysis, microbial encapsulation in monodisperse hydrogel gliadin microspheres was performed with Cellena Flow Focusing technology (Ingeniatrics). Bacterial proliferation inside the particles and its ability to degrade gliadin when growing was monitored by flow cytometry for 96 hours. The GIP content was detected by inoculating the particles with the moAb G12-FITC. The intensity of the fluorescence decreased after 24 hours in the microparticles containing the strain 2RA3. However, it was completely reduced over a 48-hour period after incubation (Fig 2).

**Fig 3:**
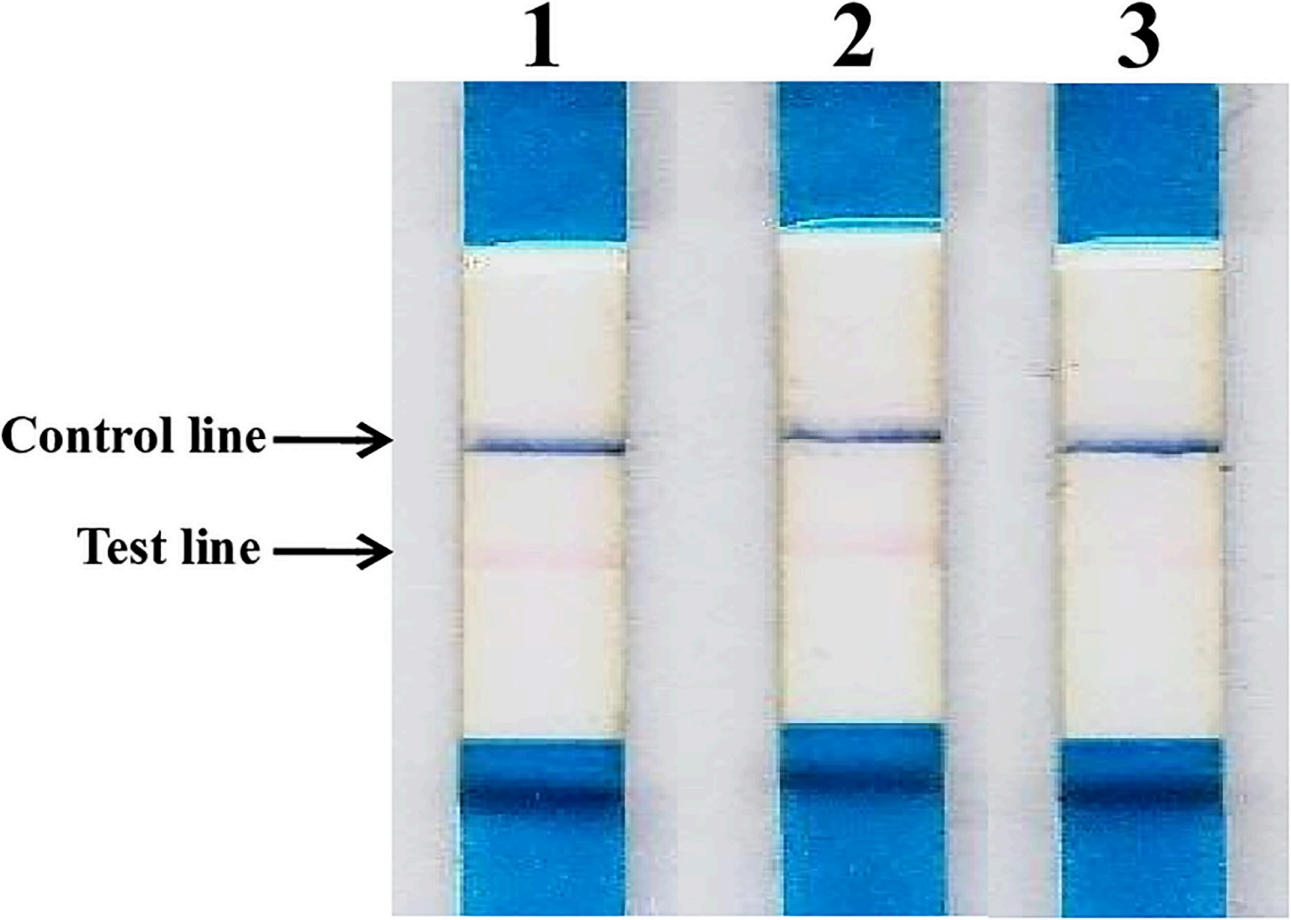
Reduction of gluten immunogenic peptides by the glutenase activity in 2RA3 cell fractions. Example of G12 immunochromatographic test containing a mixture of gliadin solution at a concentration of 90 ppm: **1**, without cellular fraction; **2**, intracellular fraction of strain 2RA3; and **3**, with extracellular fraction of strain 2RA3.

In order to determine the exact cellular location of enzymatic activity, IC strips based on G12 moAb and PWG standard gliadin solutions were used for this purpose. These IC strips provide a qualitative/semi-quantitative measure of the presence of gluten in foods or beverages, and are useful in a broad range of laboratories/industries. As indicated in Material and Methods, after fractionation of the bacterial culture, the resultant fractions were mixed with gliadin solutions and incubated overnight. The sticks were then dipped into the reaction solution for their comparison with a control without cellular fraction. A significant reduction in the intensity of the signal was observed in the extracellular fraction of strain 2RA3 as shown in Fig 3, which means that an extracellular protein is likely, responsible for the enzymatic activity.

### Microbial speciation of the strain 2RA3

**Fig 4:**
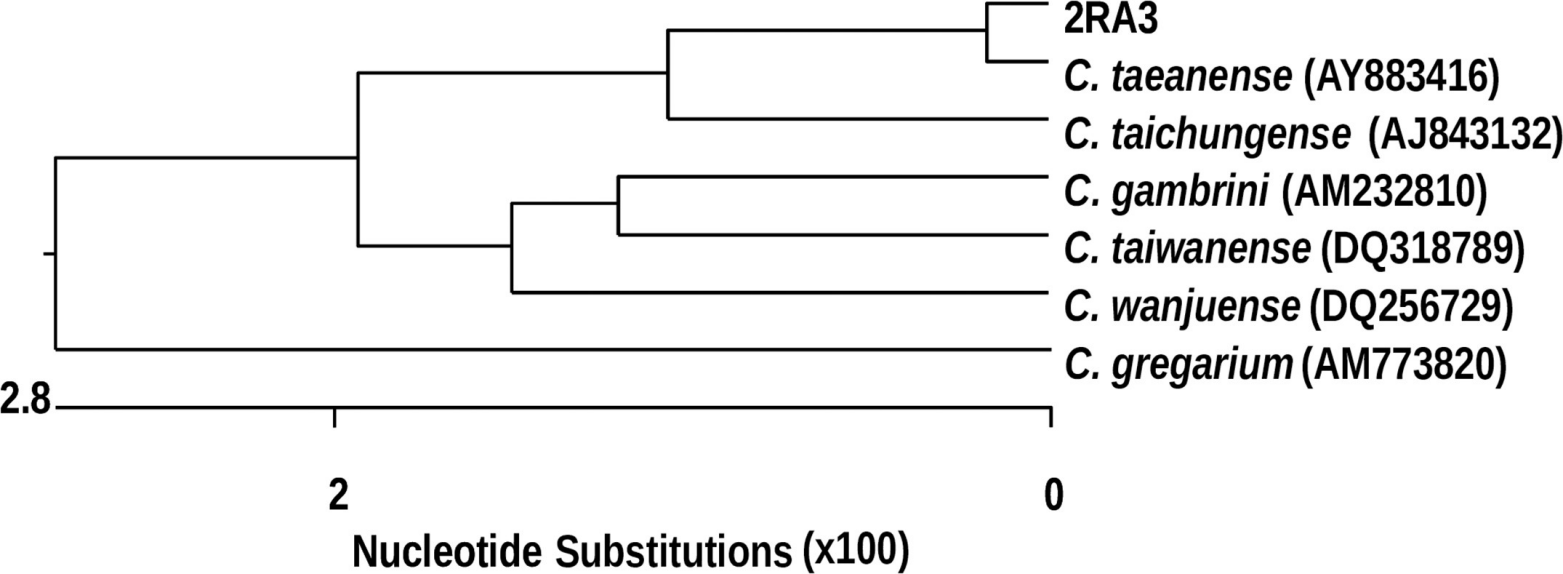
Dendrogram showing the phylogenetic relationship between strain 2RA3 and a group of related *Chryseobacterium* species.

Microbial speciation of the highest glutenase producer was first carried out through 16S rDNA analysis. The 2RA3 strain displayed the highest similarity to the genus *Chryseobacterium* (99% sequence similarity). In order to precisely define the taxonomic position of strain 2RA3 at species level, the complete 16S rRNA gene sequence was determined. Analysis of the 16S rRNA gene sequences showed that they could be placed within the phylogenetic clade encompassed by the genus *Chryseobacterium* of the family *Flavobacteriaceae*. Database searches revealed that *Chryseobacterium* sp. 2RA3 shared a sequence similarity of 99.1% with *Chryseobacterium taeanense* DSM 17071^T^ [40] and 97.9% with *Chryseobacterium taichungense* DSM 17453^T^ [41]. A phylogenetic tree was constructed using alignment sequences and similar results were obtained with various algorithms (Fig 4). The G+C content was 34.3 mol%. This value is within the calculated values, 32.1 and 36.8 mol%, obtained from the reference G+C contents of *C*. *taeanense* and *C*. *taichungense* [40, 42]. In order to elucidate a clear phylogenetic affiliation at species level, DNA-DNA hybridization experiments were necessary. Thus, DNA-DNA hybridization analysis was performed according to the method described in detail in Material and Methods. DNA-DNA hybridization values between strain 2RA3 and the type of strains with which it formed a phylogenetically coherent cluster of the genus *Chryseobacterium*, showed relatedness values of 65% with *C*. *taeanense* DSM 17071^T^ and 36% with *C*. *taichungense* DSM 17453^T^. This indicates that strain 2RA3 could constitute a new species when the recommended threshold value of 70% DNA-DNA relatedness is considered for the definition of a bacterial species by the *ad hoc* committee on reconciliation of approaches to bacterial systematics [43].

### Morphological and biochemical properties of *Chryseobacterium* sp. 2RA3

A complete characterization of the strain 2RA3 was carried out following the procedures described previously in Material and Methods. Species of the genus *Chryseobacterium* were used as a reference for comparison studies. *Chryseobacterium* sp. 2RA3 forms rod cells. Gram stain is negative. Colonies are orange-yellowish, circular, shiny, convex and 3 mm in diameter after 48 hours at 37°C. Growth occurs aerobically with 0–7% NaCl (optimal growth without salt). Good growth occurs over a broad range of pH values (6.0–10.0) and the optimal growth occurs at pH 6.0 to 7.0. The effects of various incubation temperatures on the 2RA3 growth were evaluated and the optimal growth temperature was estimated at 37°C, although growth was observed at temperatures of 28–45°C. The results showed that strain 2RA3 presented pH features typical of the species *C*. *taichungense* [41] and it showed differential pH features compared to *C*. *taeanense* [40]. A similar saline requirement was found in 2RA3 and *C*. *taeanense* [41]. The isolate 2RA3 was able to grow at 28°C and 36°C under aerobic conditions, as already reported for the *C*. *taeanense* and *C*. *taichungense* strains [40–41]. Moreover, the new isolate differed from the aforementioned *Chryseobacterium* species in terms of certain phenotypic traits (listed in Table 1). The hydrolysis of casein, gelatin and gliadin occurred in the three species compared but there was no hydrolysis of DNA and xylan. However, hydrolysis of starch and pullulan occurred in 2RA3 in contrast to the other two reference species studied. The strain 2RA3 does not hydrolyse the Tween 80 as does *C*. *taeanense* [40]. The strain 2RA3 was resistant to ampicillin, gentamicin, neomycin, kanamycin, G penicillin and chloramphenicol, but sensitive to nalidixic acid, streptomycin, amoxicillin-clavulanic acid, tetracycline and rifampicin. Assimilation and acid production from various substrates, enzyme activities, and other physiological and biochemical properties were tested using the API 20E, API 50CH and API ZYM systems. A summary of differential utilization carbon sources of strain 2RA3 and *Chryseobacterium* species is shown in S1 Table.

**Table 1:** Distinctive phenotypic characteristics of the strain 2RA3 and related species of the genus *Chryseobacterium*. Taxa: 1, *Chryseobacterium* sp. 2RA3; 2, *C*. *taeanense* DSM 17071^T^; 3, *C*. *taichungense* DSM 17453^T^. Except for the hydrolysis data of the various substrates, all data for reference species was taken from Shen [40] and Park [4,1]. ND, no data available.

Characteristic	1	2	3
**Production of**:			
**Indole**	+	-	+
**H**_**2**_**S**	-	-	-
**Acetonin**	+	-	ND
**Enzyme activities**:			
**Oxidase**	+	+	+
**Urease**	-	-	-
**ADH**	+	-	-
**LDC**	-	-	-
**CIT**	+	-	-
**TODA**	+	-	ND
**ODC**	-	ND	ND
**β-Galactosidase**	-	ND	+
**Acid production from**:			
**L-Arabinose**	+	-	-
**Fructose**	+	-	ND
**Glycerol**	-	-	ND
**Lactose**	-	-	-
**Maltose**	+	-	+
**Mannitol**	-	-	-
**Trehalose**	+	-	+
**D-Xylose**	-	-	+

Based on the phenotypic and genotypic data, the strain 2RA3 and *C*. *taeanense* DSMZ 17071^T^ [40], shares a number of phenotypic characteristics tested in common despite their differences in the following characteristics: tolerance to NaCl, pH ranges for growth, and incubation temperatures for growth. For this reason, strain 2RA3 merits recognition as a species within the genus *Chryseobacterium*, whose name is *Chryseobacterium taeanense* 2RA3.

### Gene coding *Chryseobacterium taeanense* sp. 2RA3 for glutenases: Active protein identification

The glutenase term is used to refer to enzymes that hydrolyse gluten. To the best of our knowledge, proline-specific PEPs from different sources have been largely described successfully as the enzymes responsible for glutenase activity. Analysis based on the screening in the full-length annotated genome of *C*. *taeanense* 2RA3 revealed nucleotide sequences coding for nine proteases of the S9 family. Gene-encoding putative orthologous enzymes were found in the genome of the closely related species of the genera *Chryseobacterium* (*C*. *polytrichastri*, *C*. *indologenes*, *C*. *joostei* and, *C*. *taeanense*) and with the species *Flavobacterium* sp. B17. Protein sequences D7VX69 and C6X5J5 were related to the PEP subfamily, D7VVJ1 showed high similarity to the oligopeptidase B subfamily, D7VXN3 and D7W754 with aminoacyl peptidases, while the remaining D7VY80, D7W7S6, D7W0S1 and D7VWQ8 were assigned as members of the DPP-IV family. Theoretical molecular weight and pI for the nine proteases were investigated (Table 2).

**Table 2:** *Chryseobacterium* t*aeanense* 2RA3 gene-encoding proteases of the S9 family and main characteristics of deduced translation products.

Protein sequence	GeneBank (protein) accession no.	Closest relative	% similarity	Gene length	Calculated protein molecular weight	Theoretical pI
**D7VWQ8**	SHL47965	dipeptidyl-peptidase [*Chryseobacterium polytrichastri*]	98	731	82.44	8.84
**D7W7S6**	WP_089859955	dipeptidyl aminopeptidase [*Chryseobacterium taeanense*]	100	664	75.49	7.62
**D7W0S1**	WP_074941793	dipeptidyl aminopeptidase [*Chryseobacterium indologenes*]	74	835	97.36	8.92
**D7W754**	WP_042720378	aminopeptidase P family protein [*Flavobacterium* sp. B17]	100	589	66.30	5.16
**D7VXN3**	WP_089857068	aminoacyl-histidine dipeptidase [*Chryseobacterium taeanense*]	99	480	52.43	4.92
**C6X5J5**	WP_084180263	prolyl oligopeptidase [*Chryseobacterium joostei*]	70	823	94.41	8.69
**D7VX69**	WP_089858025	prolyl oligopeptidase [*Chryseobacterium taeanense*]	98	731	78.71	7.12
**D7VVJ1**	WP_089858947	Oligopeptidase B [*Chryseobacterium taeanense*]	98	680	79.31	4.83
**D7VY80**	WP_089857180	dipeptidyl-peptidase [*Chryseobacterium taeanense*]	100	712	81.02	7.03

**Fig 5:**
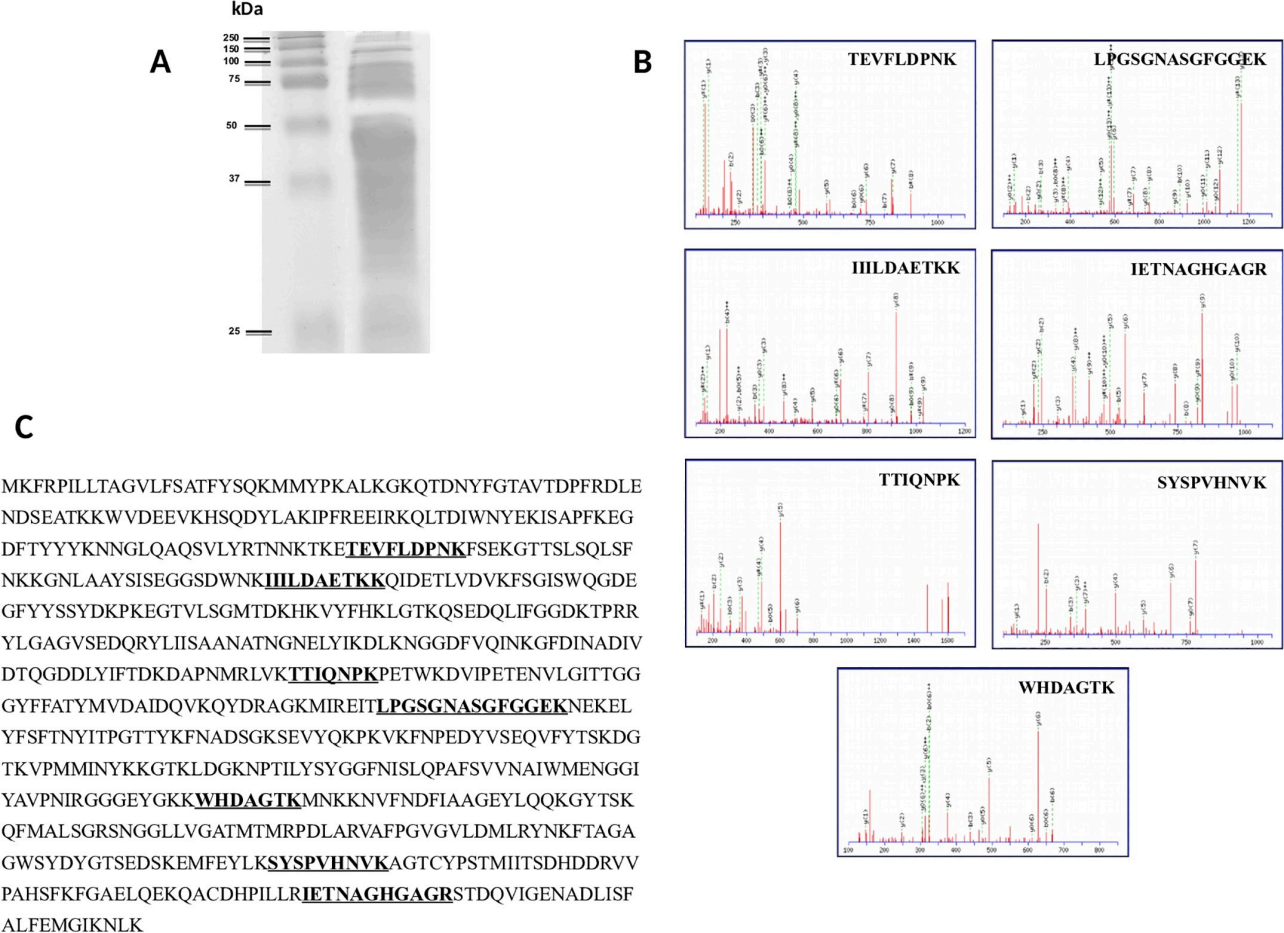
Strategy for the identification of protein responsible for the glutenase activity in *C*. *taeanense* strain 2RA3. **A**, Gliadin zymography of culture supernatant. Lane 1, molecular weight marker, lane 2, strain 2RA3. **B**, Nano-ESI-Q-TOF mass-spectrometer analysis of the potential active band after trypsin treatment; **C**, Amino acid sequence of annotated protease in the genome of *C*. *taeanense* strain 2RA3 responsible for the activity detected in the excised band.

In order to identify the enzyme responsible for the tested glutenase activity of the strain 2RA3, a gliadin zymography in the culture supernatant was performed according to the methodology described in Material and Methods. Following the electrophoretic separation of proteins, the active band of the gel corresponding to 50 kDa was excised (Fig 5A). C18 PepMap columns were employed to separate tryptic-digested proteins and then nano-ESI-Q-TOF mass spectrometer analysis was obtained. The five most intense precursors of the spectra were analysed in ion-reflector mode. The MS/MS fragmentation MASCOT analysis revealed high homology with the PEP *Flavobacterium meningosepticum*. Out of the 9 putative PEPs previously identified in the *C*. *taeanense* genome, only D7VX69 was identified. The spectra of peptides TEVFLDPNK, LPGSGNASGFGGEK, IIILDAETKK, IETNAGHGAGR, TTIQNPK, SYSPVHNVK, and WHDAGTK, which belong to protein sequence D7VX69, are shown as an illustration (Fig 5B). Therefore, this means that the D7VX69 protein sequence coding 731 amino acids was probably the PEP responsible for the activity detected in the excised band (Fig 5C). The D7VX69 prolyl endopeptidase nucleotide sequence reported in this paper has been submitted to the Bankit/GenBank database and assigned accession number MK940909 (S1 Fig).

As described in Material and Methods, the physicochemical properties of D7VX69 protein were computed by ProtParam. The theorical molecular weight and pI for this protein were 78.71 and 7.12, respectively. The extinction coefficient at 280 nm ranged from 103,600 to 103,725. The high extinction coefficient indicates the presence of a high concentration of Cys, Trp, and Tyr in the hypothetical protein [44]. The computed extinction coefficient aids in the quantitative study of protein-protein and protein-ligand interactions in the solution. The instability index value was calculated at 33.19 and, therefore, it is predicted as stable since it is smaller than the threshold of 40. A high aliphatic index indicates that a protein is thermo-stable over a wide temperature range [45]. However, the aliphatic index involved in this study was calculated at 67.52, and therefore does not share this property. D7VX69 protein in this study had a GRAVY index of -0.558. This low GRAVY range indicates the possibility of it being a globular (hydrophilic) protein rather than membranous (hydrophobic) [46]. The total number of negatively and positively charged residues were 88 and 86, respectively. Therefore, there were no significant differences and it supposes a net charge.

### Expression, purification, and characterization of the recombinant prolyl endopeptidase of *Chryseobacterium taeanense* sp. 2RA3

**Fig 6:**
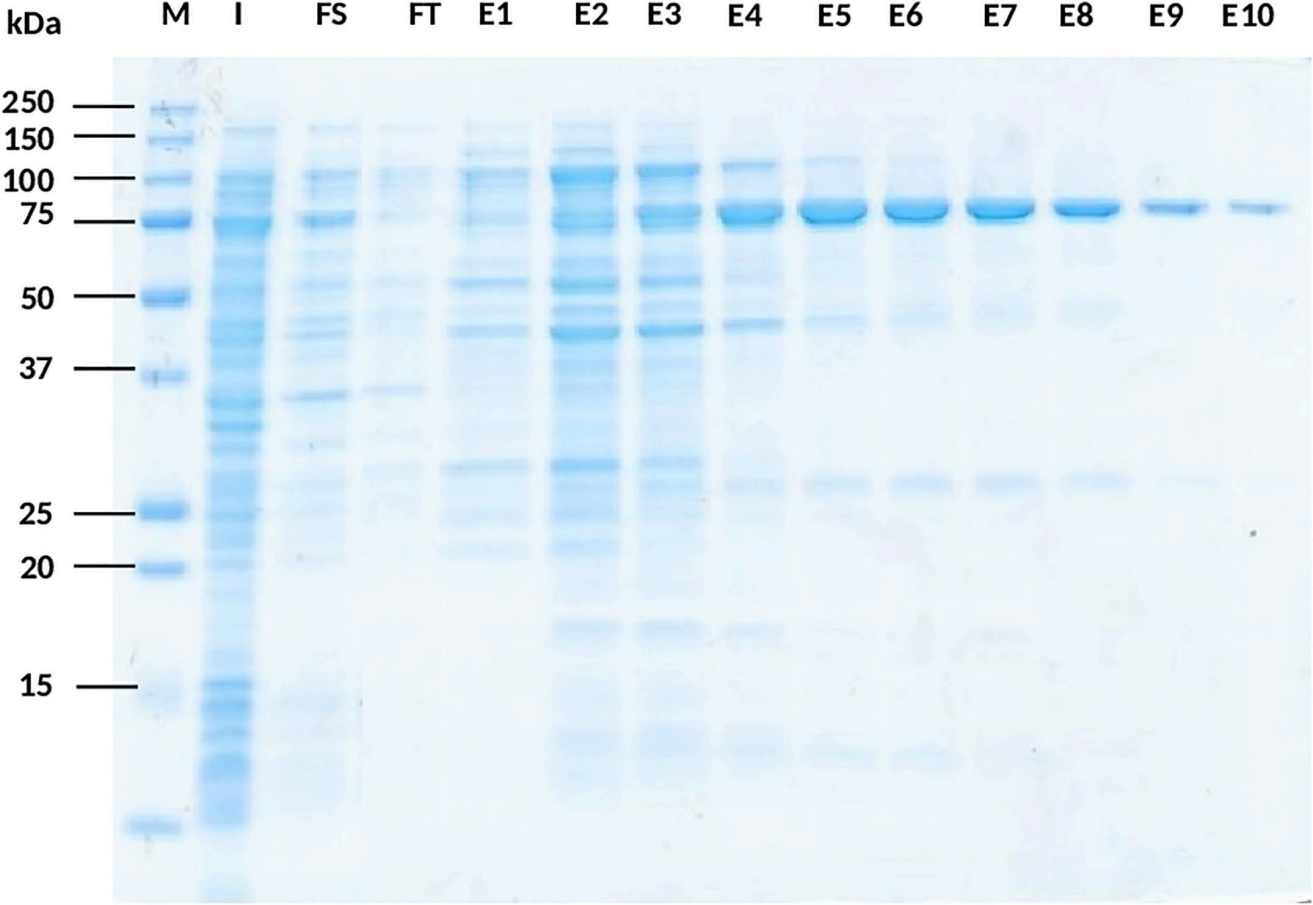
Purification of PEP 2RA3 from *E*. *coli* REG-1 [pALEX2-HCa]. SDS-PAGE was Coomassie stained. **M**, Molecular weight marker; **I**, Fraction of *E*. *coli* induced culture before purifying; **FS**, Solubilized fraction; **FT**, Flow-through fraction from Ni-NTA resin; **E1-E10**, Elution fractions of desired protein at different concentrations of imidazole (25 mM -500 mM).

Protein sequence D7VX69 was chosen from the investigated entries as being responsible for the PEP activity after gliadin zymography check and analysis of excised band by mass-spectrometer. The full-length gene-encoding PEP 2RA3 was inserted into a vector with a polyhistidine tag, expressed in *E*. *coli*, and the expression was induced by 1 mM salicylate and 10 mM methylbenzoate at 20°C. When compared to the sample without induction, only the induced cells containing the recombinant vector overexpressed an extra 80 kDa protein. The recombinant PEP was purified with Ni-NTA resin as described in the Material and Methods section. The purified recombinant enzyme migrated as a single band on 12.5% SDS-PAGE, similar to that of the calculated MW of 80.29 kDa (Fig 6). The discrepancy occurred in the a molecular weight of the recombinant prolyl endopeptidase purified and the hydrolysis signal in the zymogram is due to the PCR-amplified product contain an extra C-terminally histidine tag and secondly, the native conditions in which zymogram is performed mask the real molecular weight of certain proteins.

**Fig 7:**
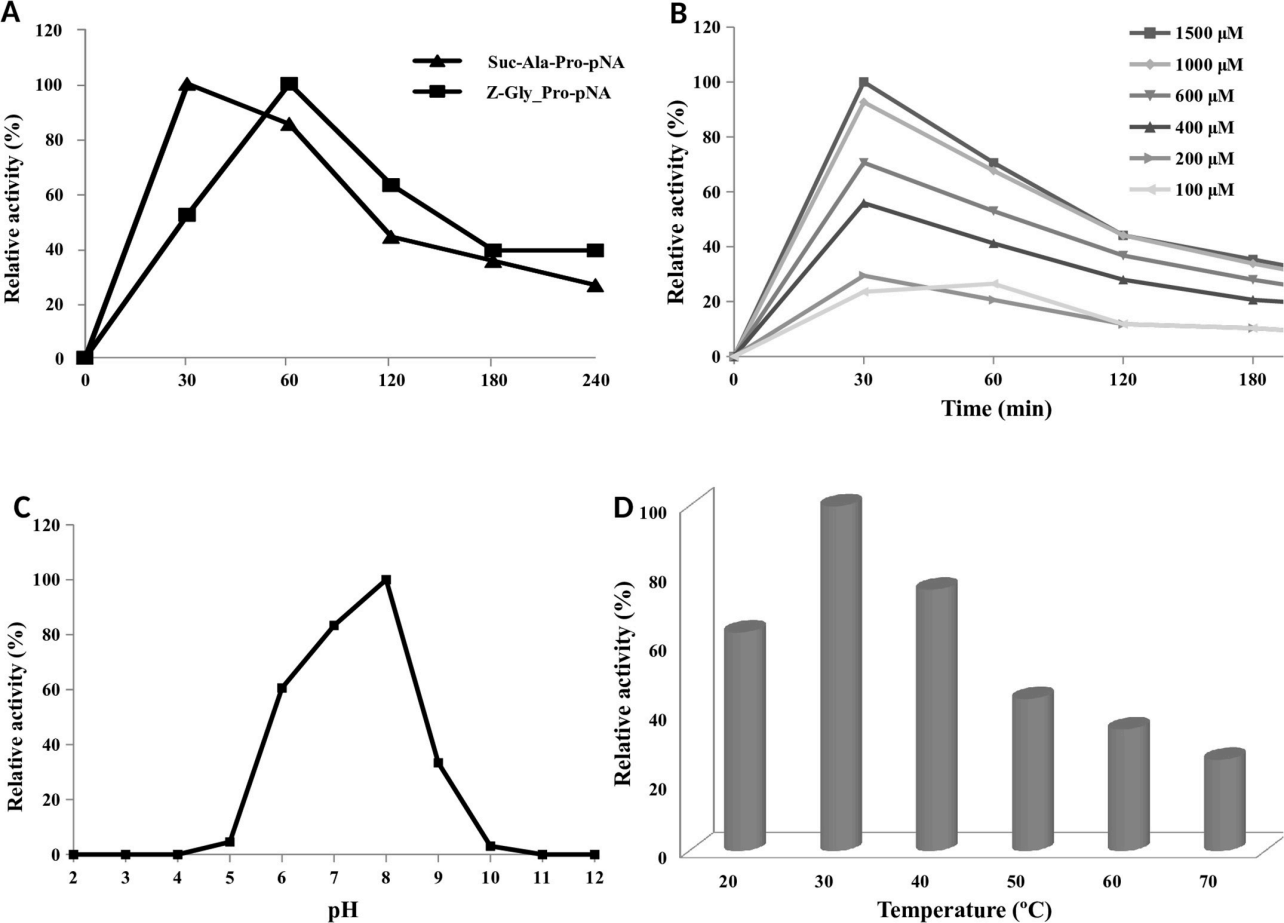
Characterization of the recombinant PEP. **A**, Hydrolysis of Suc-Ala-Pro-pNA and Z-Gly-Pro-pNA by recombinant PEP. **B**, Activity profiles measured using Suc-Ala-Pro-pNA at various concentrations. **C**, The effect of pH was determined by incubating the enzyme in citrate/disodium phosphate buffer (pH 2–8), Tris-HCl (9–10), and glycine/NaOH (pH 11–12) for 30 min with 1.5 mM Suc-Ala-Pro-pNA. **D**, Hydrolysis of 1.5 mM Suc-Ala-Pro-pNA at various temperatures. The activity for optimum temperature was determined under conditions at 20–70°C and optimum pH for 30 min.

The functionality of the PEP was confirmed by its ability to hydrolyse synthetic chromogenic peptide substrates. The release of the intramolecularly quenched fluorescence after hydrolysis of synthetic peptides containing the pNA group was investigated. The optimal conditions for the PEP enzyme in terms of activity and stability were examined using Z-Gly-Pro-pNA and Suc-Ala-Pro-pNA as substrates. As observed in Fig 7A, the enzyme hydrolysed both substrates after 30–60 minutes of reaction. Although Z-Gly-Pro-pNA was insoluble at higher concentrations, preventing kinetic measurements under substrate-saturated conditions, the substrate was effective in detecting enzyme activity in minor concentrations (100–200 μM). On the contrary, Suc-Ala-Pro-pNA had high water solubility at all pH values tested and therefore was selected as preferred substrate for kinetic studies. The optimal Suc-Ala-Pro-pNA was determined at 1000 μM (Fig 7B) and the optimal enzyme concentration to carry out the reaction was 0.2 μM; however, the enzyme retained 80% of its activity with 0.02 μM.

In order to determine the pH optimum of PEP 2RA3, the enzyme was incubated with Suc-Ala-Pro-pNA in buffers spanning the pH range of 2–12 and the activity was measured (Fig 7C). According to the pH profile, the enzyme showed the highest activity at pH 8.0, and over 50% of the activity was retained between pH 6.0 and 9.0. Earlier studies showing various alkaline pH optima and pH stability exhibited by PEPs were reported. PEP isolated from *Halobacterium halobium* S9, *Sphingomonas capsulate* and *Pseudomonas* sp. KU-22 showed pH optimal at 8.7, 8.5 and 8.0, respectively [47–49]. In addition, *Xanthomonas* sp. and *Aeromonas hydrophila* had optimal activity at pH 7.7 [50–51] and *Flavobacterium meningosepticum* had its highest activity at pH 7.0 [52]. Conversely, other PEPs as *Aspergillus oryzae*, *Aspergillus niger* and *Sphaerobacter thermophiles* achieved the maximum activity at the acidophilic pH values 4.0, 4.2 and 6.6, respectively [14, 53–54].

The thermal stability of the enzyme is also depicted in Fig 7D. The PEP was stable at up to 40°C, with a relative activity of over 75% incubating at pH 8.0 for 1 hour. The optimum temperature for the PEP was established at 30°C, whereas a decrease in activity was observed when the enzyme was heated at temperatures above 60°C. At higher temperatures, the PEP probably underwent denaturation and lost its activity. Previous studies of PEPs showed temperature optima ranging from 30°C to 50°C. The optimal temperature of the PEP from *A*. *hydrophila* was also reported to be 30°C [51]. However, *A*. *niger* and *Pseudomonas* sp. KU-22 showed optimal temperatures of 42°C and 45°C, respectively [49, 54].

According to Verhoeckx et al. [55], acid hydrolyses and heating at high temperature in gluten-containing cereals such as wheat may enable the protein aggregation resistant to digestibility and with allergenic potential so that specific proteolytic properties are needed. Therefore, the optimal conditions for the PEP 2RA3 action make it a promising enzyme for its application to gluten food processes.

### Effect of PEP in beer gluten peptides

Apart from their above mentioned pharmaceutical and medicinal applications [56–57], an important role of PEPs in the production of various foodstuffs has been reported. Specifically, PEPs have been proposed needed for an efficient degradation of gluten in raw material or during food processing [58].

In brewing beer it is necessary malt, mainly from barley and/or wheat containing proline and glutamine-rich proteins, brewing water, raw hops and top-or bottom-fermenting yeast. Because many proteins are degraded and/or otherwise modified due to differences in the brewing process, a huge diversity of gluten content in the different beers is found despite the majority maintain high gluten levels [37].

It is well known that PEPs cleave peptide bonds on the C-terminal side of prolyl residues within peptides that are up to approximately 30 amino acids long. Research published by Van Landschoot [59] reported that 100%-barley malt beers could be rendered gluten-free using PEP. AN-PEP is the only proline-specific PEP that is industrially available and can cope with the acidic conditions of beer fermentation [60]. However, a number of barley-based beers that are labelled “crafted to remove gluten” have recently become commercially available [61].

**Fig 8:**
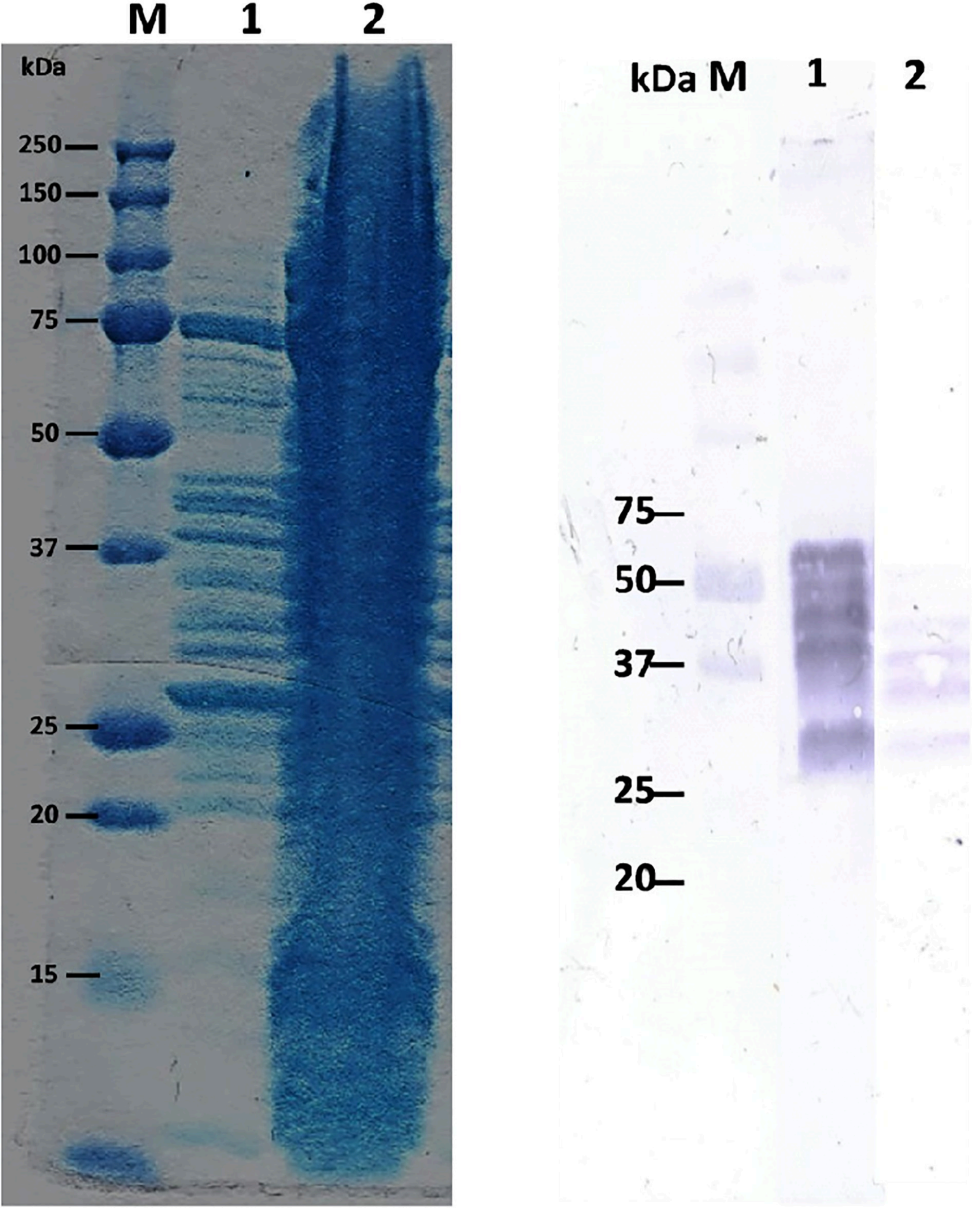
Effect of PEP in beer gluten peptides. **A**. Coomassie stained protein gel. **M**, Molecular weight marker; **1**, Beer fraction after addition of PEP showing the hydrolysis of gluten beer peptides by the purified recombinant PEP. **2**, Beer fraction. **B**. Western blots using the detector antibody G12. **M**, Molecular weight marker; **1**, Beer fraction; **2**, Beer fraction after addition of PEP.

In order to determine whether the recombinant PEP 2RA3 acts on beer gluten peptides by modifying their band pattern, we performed an assay with a commercial beer with a gluten content of approximately 100 ppm. The electrophoresis pattern following Coomassie staining after overnight incubation is shown in Fig 8A. We proved that a part of the peptides in gluten beer is hydrolysed to smaller peptides by PEP 2RA3. Western blot analysis with the anti-GIP moAb was further assayed to determine the variations in celiac immunotoxicity of peptides remaining after PEP addition. As observed in Fig 8B, a large reduction of GIP was detected in the beer treated with the PEP although incomplete neutralization of its antigenic epitopes occurred. According to results in Fiedler [62], PEP addition led to a reduction in the amount of intact gluten, however; PEP treatment failed to completely degrade all known immunogenic sequences. Fewer gluten peptides containing immunogenic sequences were detected in the PEP-containing beer in the presence of up to 6 times the manufacturer’s recommended dosage of PEP.

Numerous dietary supplements already on the market are marketed to aid in the digestion of gluten and reduce its toxicity. Clinical studies investigating the effect of glutenases on symptoms and biomarkers in CD patients is unfortunately not as straightforward as it might seem [19, 63]. Not all promising enzymes have been tested in vivo. Because not all enzymes are equally efficient, mainly in large dose-ranging studies, and due to their specificity, it is generally used a combination of different enzymes designed "enzymatic cocktail" in the different food and beverage biotechnological processes.

## Conclusions

The present study reveals a highly active prolyl-endopeptidase from *Chryseobacterium taeanense* that exhibits specificities of interest when targeting immunogenic gluten domains. It is one of the few PEP enzymes that have been confirmed to have such GIP reduction activity. When expressed in *Escherichia coli* and isolated, this prolyl-endopeptidase was found to have basic pH stability and ambient temperature activity and stability, which renders it a promising enzyme for the gluten food process applications needed for the reduction of the allergenicity of wheat products. Concretely, we consider that PEP 2RA3 should combine with other specific enzymes to reach complete elimination of GIP in raw material or during food processing, since GIP cannot remain in final product to be suitable and safe for celiac.

## Supporting information

S1 Table API ZYM profiles of *Chryseobacterium* strain 2RA3 and other species of the genus *Chryseobacterium*. Taxa: 1, *Chryseobacterium* sp. 2RA3; 2, *C*. *taeanense* DSMZ 17071^T^; 3, *C*. *taichungense* DSMZ 17453^T^. Data for taxa 2 are from Park [40]. Data for taxa 3 are taken from Shen [39]. All taxa showed positive reactions for 2-Naphthyl butyrate, 2-Naphthyl-αD-glucopyranoside and 6-Br-2-Naphthyl-βD-glucopyranoside. All taxa showed negative reactions for 6-Br-2-Naphthyl-αD-galactopyranoside and 2-Naphthyl-αL-fucopyranoside. The intensity of the colour was measured on a scale from 0 to 5, and interpreted as negative (–) when values ranged from 0 to 1 and positive (+) for values ranging from 2 to 5.
(DOCX)

S1 Fig D7VX69 prolyl endopeptidase nucleotide sequence. (DOCX)
