# Supplementary material for: A new microbial gluten-degrading prolyl endopeptidase: Potential application in celiac disease to reduce gluten immunogenic peptides
Source: PLoS One. 2019 Jun 27;14(6):e0218346. doi: 10.1371/journal.pone.0218346 (PMC6597064; doi:10.1371/journal.pone.0218346)
Supplement: S1 Table — Taxa: 1, Chryseobacterium sp. 2RA3; 2, C. taeanense DSMZ 17071T; 3, C. taichungense DSMZ 17453T. Data for taxa 2 are from Park [40]. Data for taxa 3 are taken from Shen [39]. All taxa showed positive reactions for 2-Naphthyl butyrate, 2-Naphthyl-αD-glucopyranoside and 6-Br-2-Naphthyl-βD-glucopyranoside. All taxa showed negative reactions for 6-Br-2-Naphthyl-αD-galactopyranoside and 2-Naphthyl-αL-fucopyranoside. The intensity of the colour was measured on a scale from 0 to 5, and interpreted as negative (–) when values ranged from 0 to 1 and positive (+) for values ranging from 2 to 5. (DOCX) [file pone.0218346.s001.docx]

**S1 Table.** API ZYM profiles of *Chryseobacterium* strain 2RA3 and other species of the genus *Chryseobacterium.* Taxa: 1, *Chryseobacterium* sp. 2RA3; 2, *C. taeanense* DSMZ 17071^T^; 3, *C. taichungense* DSMZ 17453^T^. Data for taxa 2 are from Park [40]. Data for taxa 3 are taken from Shen [39]. All taxa showed positive reactions for 2-Naphthyl butyrate, 2-Naphthyl-αD-glucopyranoside and 6-Br-2-Naphthyl-βD-glucopyranoside. All taxa showed negative reactions for 6-Br-2-Naphthyl-αD-galactopyranoside and 2-Naphthyl-αL-fucopyranoside. The intensity of the colour was measured on a scale from 0 to 5, and interpreted as negative (–) when values ranged from 0 to 1 and positive (+) for values ranging from 2 to 5.

| **Substrate** | **1** | **2** | **3** |
| --- | --- | --- | --- |
| **2-Naphthyl butyrate** | + | + | + |
| **2-Naphthyl myristate** | - | - | + |
| **L-Cystyl-2-naphthylamide** | - | - | + |
| **N-Benzoyl-DL-arginine 2-naphthylamide** | - | - | + |
| **N-Glutaryl-phenylalanine 2-naphthylamide** | - | - | + |
| **6-Br-2-Naphthyl-αD-galactopyranoside** | - | - | - |
| **2-Naphthyl-βD-galactopyranoside** | - | - | ND |
| **Naphthol-AS-BI-βD-glucuronide** | - | - | + |
| **2-Naphthyl-αD-glucopyranoside** | + | + | + |
| **6-Br-2-Naphthyl-βD-glucopyranoside** | - | + | + |
| **1-Naphthyl-N-acetyl-βD-glucosaminide** | - | - | + |
| **2-Naphthyl-αL-fucopyranoside** | - | - | - |
