# Supplementary material for: A new microbial gluten-degrading prolyl endopeptidase: Potential application in celiac disease to reduce gluten immunogenic peptides
Source: PLoS One. 2019 Jun 27;14(6):e0218346. doi: 10.1371/journal.pone.0218346 (PMC6597064; doi:10.1371/journal.pone.0218346)
Supplement: S1 Fig — (DOCX) [file pone.0218346.s002.docx]

**S1 Fig.** D7VX69 prolyl endopeptidase nucleotide sequence.

> D7VX69

1 atgatgtatc ccaaagcatt aaaaggaaag caaaccgata attattttgg aactgctgtt

61 acagatccgt tcagagatct tgaaaatgat tctgaagcca ccaaaaagtg ggtcgacgaa

121 gaagtgaaac acagtcagga ttatcttgca aaaatccctt tcagagaaga aatcaggaaa

181 cagctcaccg atatctggaa ctacgaaaaa atttcggctc cttttaaaga aggtgatttt

241 acttattatt ataaaaacaa cgggctacag gcacaatctg tactttacag aaccaacaat

301 aaaacgaaag aaacagaagt atttttagat ccgaataaat tttctgaaaa aggcaccact

361 tcactttctc aattgtcttt taacaaaaaa ggaaatctcg ccgcttattc tatttcagaa

421 ggaggaagtg actggaacaa gatcattatc atagacgctt tatctaaaaa acagatcgat

481 gaaacgctgg tagatgtaaa gttcagcgga atttcctggc agggtgatga aggtttctat

541 tattcaagct acgacaaacc gaaagaggga accgtgctct ccggcatgac cgataagcat

601 aaagtctatt ttcataagtt aggaacaaag cagtccgaag atcaattgat ttttggagga

661 gataaaacac cgagaagata tttgggagca ggagtgtctg aagatcagag atatcttatt

721 atttctgctg cgaatgccac caacggaaat gaattataca taaaagacct taaaaacgga

781 ggagattttg tgcagattaa taaaggtttt gatatcaatg ccgatatagt cgatacacaa

841 ggagacgatc tgtatatctt taccgataag gatgccccga atatgcgtct cgtaaaaaca

901 accattaaaa atcctgctcc agagacttgg aaagatgtaa ttccggaaac ggaaaatgtt

961 ttgggaatca caacaggcgg aggatatttc tttgctacct atatggtgga tgcgattgat

1021 caggtaaaac aatacgacag agcaggaaaa atgatccgtg aaattacgct tcccggaaaa

1081 ggaaatgttt ctggttttgg aggaaaggaa aacgagaaag aattgtattt ttcattcacc

1141 aattatatta caccgggaac aacgtataaa ttcaatgcag attccggaaa atctgaggtt

1201 taccagaagc cgaaggtgaa atttaatcct gaagattatg tctctgaaca agtattttac

1261 acctcaaaag acggtacaaa agttccgatg atgattaact ataaaaaagg aactaagctc

1321 gacggtaaaa atcctacgat tttatattct tacggaggtt ttaatatcag tttgcagccg

1381 gccttctctg tagtcaatgc catctggatg gaaaacggtg gtatttatgc cgttccgaac

1441 atccgtggag gtggtgaata tggaaaaaaa tggcatgatg ccggaacaaa aatgaataag

1501 aaaaacgtat tcaacgattt catcgctgcc ggagaatatt tgcagcagaa aggctacact

1561 tccaaacagt ttatggctct ttcgggaaga tcaaatggag gactgctggt gggtgcaaca

1621 atgacgatgc gtcccgatct ggcaagagtc gctttcccgg gagttggcgt gttggatatg

1681 ctgaggtata ataaattcac agccggtgca ggttggtcgt acgattacgg aacctctgaa

1741 gatagcaaag aaatgtttga atatttaaaa tcatattccc cggttcataa tgtaaaagcc

1801 ggaacatgct atccttccac aatgattatc accagcgatc atgatgacag ggtggttcct

1861 gcgcattctt tcaaattcgg agctgagctt caggaaaagc aggcatgcga tcatccgatt

1921 cttttaagga ttgaaaaaaa tgcaggtcac ggagcaggca gatctacaga tcaggtgatc

1981 ggagaaaatg cagacttgat ttctttcgct ttatttgaaa tggggattaa aaatttaaag
